# Parametric models for biomarkers based on flexible size distributions

**DOI:** 10.1002/hec.3787

**Published:** 2018-06-14

**Authors:** Apostolos Davillas, Andrew M. Jones

**Affiliations:** ^1^ Institute for Social and Economic Research University of Essex Colchester UK; ^2^ Department of Economics and Related Studies University of York York UK; ^3^ Centre for Health Economics Monash University Melbourne Australia

**Keywords:** biomarkers, generalized beta of second kind, heavy tails, tail probabilities

## Abstract

Recent advances in social science surveys include collection of biological samples. Although biomarkers offer a large potential for social science and economic research, they impose a number of statistical challenges, often being distributed asymmetrically with heavy tails. Using data from the UK Household Panel Survey, we illustrate the comparative performance of a set of flexible parametric distributions, which allow for a wide range of skewness and kurtosis: the four‐parameter generalized beta of the second kind (GB2), the three‐parameter generalized gamma, and their three‐, two‐, or one‐parameter nested and limiting cases. Commonly used blood‐based biomarkers for inflammation, diabetes, cholesterol, and stress‐related hormones are modelled. Although some of the three‐parameter distributions nested within the GB2 outperform the latter for most of the biomarkers considered, the GB2 can be used as a guide for choosing among competing parametric distributions for biomarkers. Going “beyond the mean” to estimate tail probabilities, we find that GB2 performs fairly well with some disparities at the very high levels of glycated hemoglobin and fibrinogen. Commonly used linear models are shown to perform worse than almost all the flexible distributions.

## 1 INTRODUCTION

Recent developments in social surveys include the integration of biomarkers and self‐reported health measures. Biomarkers are objectively measured indicators of normal biological or pathogenic processes and, as such, offer at least two key advances over self‐report health. First, biomarkers are not subject to reporting bias; given evidence for socio‐economic‐related reporting bias in health, biomarkers offer a significant advantage in socioeconomic inequalities research (Bago d'Uva, O'Donnell, & van Doorslaer, 2008; Carrieri & Jones, 2017). Second, biomarkers can contribute to our understanding of the underlying biological factors through which socioeconomic conditions get “under the skin” (e.g., thought stress‐related physiological responses) and the role of socioeconomic exposures at earlier pre‐symptomatic health states (Davillas, Benzeval, & Kumari, 2017; Jürges, Kruk, & Reinhold, 2013).

A growing literature analyses the effect of socioeconomic position on the conditional mean of biomarkers (e.g., Davillas et al., 2017, Jürges et al., 2013). However, biomarkers create several statistical modeling challenges as they often have skewed distributions with heavy tails (Jones, 2017). Furthermore, errors are likely to be heteroskedastic and responses to covariates may be nonlinear. Existing studies have estimated linear regression models using ordinary least squares (OLS) on raw or log‐transformed biomarkers (Jürges et al., 2013) and alternative inherently nonlinear specifications, such as the generalized linear models (Davillas et al., 2017). Although OLS on log rather than on levels might improve performance by reducing skewness, re‐transformation to the raw scale—as health policymakers require—is highly challenging, requiring knowledge of the degree and form of heteroscedasticity (Jones, Lomas, & Rice, 2014). Although the generalized linear model family deals with heteroskedasticity, it fails to explicitly account for skewness and kurtosis, imposing potential bias and efficiency losses (Jones et al., 2014).

Our paper contributes to the literature on modeling biomarkers by comparing the performance of a set of more flexible parametric distributions, the generalized beta of the second kind (GB2), the generalized gamma (GG), and their nine nested and limiting cases; we use nationally representative UK data on commonly used blood‐based biomarkers for inflammation, diabetes, cholesterol, and stress‐related hormones (Carrieri & Jones, 2017). The GG and GB2 allow for a wide range of skewness and kurtosis to better accommodate the biomarker data generation processes; these distributions have been proposed for fitting heavily skewed outcomes (e.g., health care costs; Jones et al., 2014), to which biomarkers share similar distributional features. For comparison purposes, linear regression models using OLS are also estimated. Given that different biomarkers exhibit different distributions, identifying GB2 as a discriminatory tool among competing distributions might be useful for health researchers. Going “beyond the mean”, we also explore to what extent the GB2 and its nested cases that exerted the best goodness of fit (for each biomarker) regarding the whole distribution also perform well to predict tail probabilities.

## 2 METHODS

The three‐parameter GG distribution has been introduced as robust alternative to common estimation techniques for asymmetric data (Manning, Basu, & Mullahy, 2005). More recently, Jones, Lomas, and Rice (2014) have suggested adding further flexibility based on the four‐parameter GB2 distribution. GB2 allows for a wider range of skewness and kurtosis, choosing among its several special or nested cases, whereas GB2's extra flexibility may also enhance performance (Jones et al., 2014).

The GG distribution has a density function and conditional expectation that take the form:

(1) $$f\left(y;\kappa ,\mu ,\sigma \right)=\frac{\gamma^{\gamma }}{\sigma y\sqrt{\gamma }\Gamma \left(\gamma \right)}\exp\left(z\sqrt{\gamma }-u\right)$$

and

(2) $$E\left(y|x\right)=\exp\left(x^{\prime }\beta \right)\left[\kappa^{2\sigma /\kappa }\frac{\Gamma \left(\frac{1}{\kappa^{2}}+\frac{\sigma }{\kappa }\right)}{\Gamma \left(\frac{1}{\kappa^{2}}\right)}\right],$$

where *γ* = |*κ*|^−2^, *z* =  *sign* (κ){ln(*y*) − μ}, *u* = *γ* exp (|κ|*z*), μ = *x*^′^β, and Γ(.) is the gamma function. Parameters κ and σ are the shape parameters (Manning et al., 2005). The GG nests the gamma (κ = σ), Weibull (κ = 1), exponential (κ = 1, σ = 1), and lognormal (κ = 0) distributions.

The four‐parameter GB2 distribution adds further flexibility and has a probability density function and conditional mean of

(3) $$f\left(y;a,b,p,q\right)=\frac{ay^{\mathrm{ap}-1}}{b^{\mathrm{ap}}B\left(p,q\right){\left[1+{\left(\frac{y}{b}\right)}^{a}\right]}^{\left(p+q\right)}}$$

and

(4) $$E\left(y|x\right)=b\left[\frac{\Gamma \left(p+\frac{1}{a}\right)\Gamma \left(q-\frac{1}{a}\right)}{\Gamma \left(p\right)\Gamma \left(q\right)}\right],$$

where *b* =  exp (*x*^′^β) and B(.) and Γ(.) are the beta and gamma functions (Jones et al., 2014). Parameter *a* influences kurtosis and *p* and *q* the skewness of the distribution. We also estimate the nested and limiting cases of GB2: the three‐parameter beta of the second kind (B2) [*a* = 1], Singh–Maddala (SM) [*p* = 1], and Dagum [*q* = 1]; the two‐parameter Fisk [*p* = *q* = 1], and Lomax [*p* = *a* = 1]. GG itself is also a limiting case of the GB2, where *b* = *q*^1/*a*^β and *q* → ∞ (Jones et al., 2014). We also estimate linear regression models using OLS.

The restrictions imposed by each of the special and limiting cases within the GG and GB2 are evaluated using Wald and likelihood ratio (LR) tests. To assess the comparative performance of beta‐ with gamma‐family distributions (being limited cases and not a linear restriction of a parameter), we compare Akaike (AIC) and Bayesian (BIC) information criteria across all models (Jones et al., 2014).

## 3 DATA

The UK Household Panel Study (UKHLS) is a large, nationally representative UK study. At UKHLS Wave 2, participants from its predecessor, the British Household Panel Survey, were also incorporated. Non‐fasted blood samples were collected, after the UKHLS Wave 2 interview for the original UKHLS respondents and, at Wave 3, for the British Household Panel Survey sample. Pooling biomarker data from UKHLS Waves 2 and 3 (2010–2013) resulted in a potential sample of 13,107 respondents.

**Figure A1:**
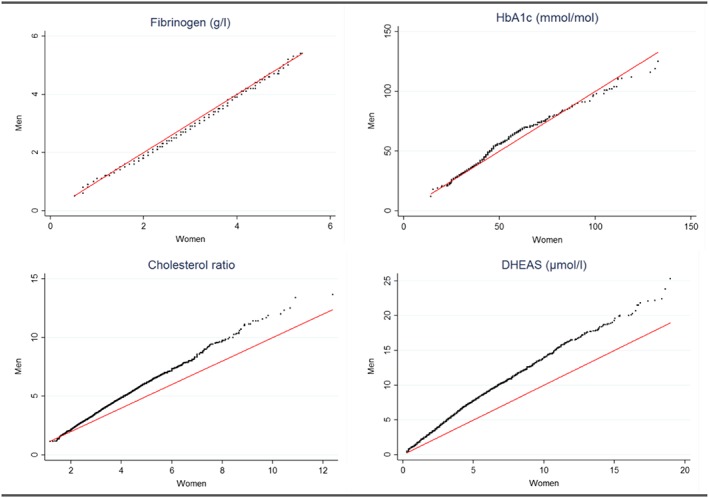
Quantile–quantile plots of the biomarkers by gender [Colour figure can be viewed at http://wileyonlinelibrary.com]

Four biomarkers are used. Fibrinogen is an inflammatory biomarker, with higher values linked to cardiovascular morbidity and all‐cause mortality risks (Davillas et al., 2017). Glycated hemoglobin (HbA1c) is a diagnostic biomarker for diabetes. The ratio of total cholesterol to high‐density lipoprotein cholesterol is used as a marker for fatty substances in the blood. Dehydroepiandrosterone sulfate (DHEAS) is a steroid hormone and one of the mechanisms through which psychosocial stressors might affect health (Vie, Hufthammer, Holmen, Meland, & Breidablik, 2014). Given our focus on the comparative performance of parametric distributions regarding goodness of fit, rather than explore potential effects from covariates, a parsimonious set of covariates is used: polynomials of age (cubic or quartic depending on the biomarker used), gender, and their interactions to allow for flexible gender effects (Figure A1). 1The limited number of covariates may also alleviate concerns that, for less parsimonious specifications, the best specification for each model need to be compared rather than using the same covariates (Jones et al., 2014). However, the relative performance of our models (Table 2) remained the same in the case of no covariates.


## 4 RESULTS

**Figure 1:**
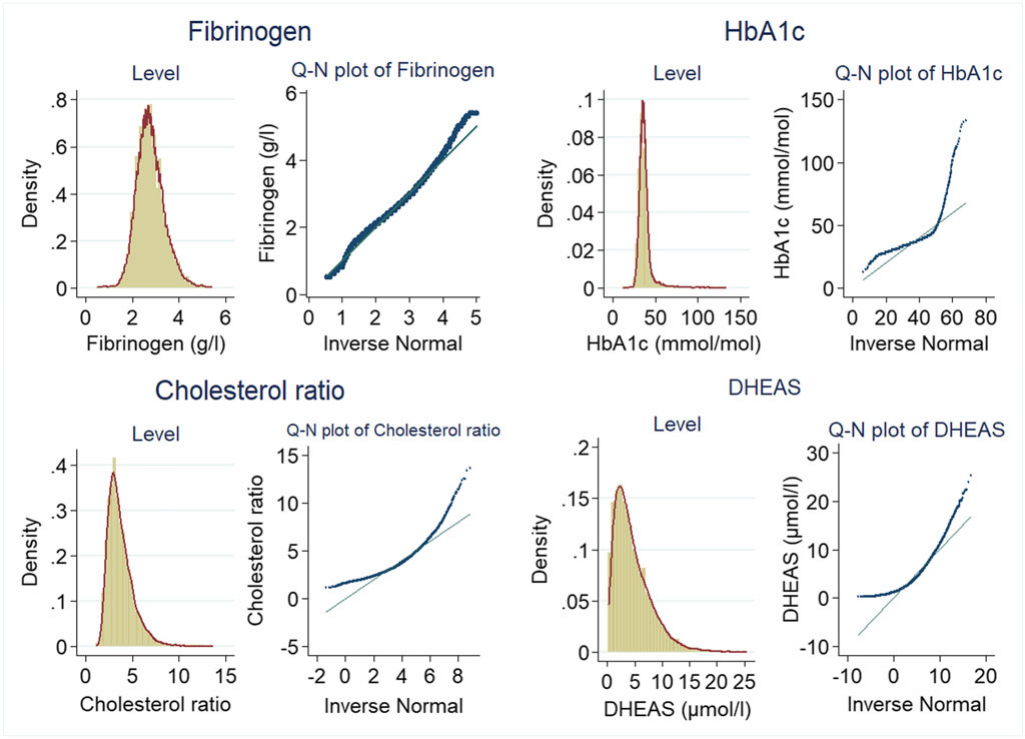
Distribution of biomarkers and quantile‐normal (Q‐N) plots. DHEAS: dehydroepiandrosterone sulfate; HbA1c: glycated hemoglobin [Colour figure can be viewed at http://wileyonlinelibrary.com]

Figure 1 presents the distribution of biomarkers (descriptive statistics in Table A1). Fibrinogen has a symmetric distribution but with fat tails (Figure 1). HbA1c is much more skewed (skewness statistic of 4.2 compared with zero for normal data) with long right‐hand tails and excess kurtosis (31.15 vs. 3 for normal data; Table A1). The cholesterol ratio and DHEAS also exhibit long right‐hand tails and high kurtosis.

Table 1 contains restriction tests for the nested and limiting cases within the GG and GB2. Across all biomarkers, we find no evidence in support of any of the special cases within the GG distribution. For fibrinogen, we are unable to reject the null hypothesis of the restriction being valid for the SM model. Our results for HbA1c do not support any of the nested distributions. For the cholesterol ratio, both the LR and Wald tests favor the B2 distribution. Although the Wald test also fails to reject the null hypothesis for SM, this is not confirmed by the LR test; this disparity reflects the wide confidence intervals for GB2's *p* parameter (which include both 1, satisfying the SM restriction, and 0; Table A2). Our results for DHEAS favor the SM distribution.

**Table 1:** LR and Wald tests (*p*‐values) for special cases of the GB2 and GG

	Fibrinogen		HbA1c		Cholesterol ratio		DHEAS	
	LR	Wald	LR	Wald	LR	Wald	LR	Wald
GB2 versus …								
B2	0.000	0.000	0.000	0.000	**0.247**	**0.193**	0.000	0.000
SM	**0.208**	**0.236**	0.000	0.000	0.000	0.188	**0.703**	**0.710**
Dagum	0.004	0.013	0.000	0.000	0.000	0.020	0.000	0.000
Fisk	0.002	0.000	0.000	0.000	0.000	0.000	0.000	0.000
Lomax	0.000	0.000	0.000	0.000	0.000	0.000	0.000	0.000
GG versus …								
Gamma	0.000	0.024	0.000	0.000	0.000	0.000	0.000	0.000
Lognormal	0.000	0.000	0.000	0.000	0.000	0.000	0.000	0.000
Weibull	0.000	0.000	0.000	0.000	0.000	0.000	0.000	0.000
Exponential	0.000	0.000	0.000	0.000	0.000	0.000	0.000	0.000

*Note*. B2: beta of the second kind; DHEAS: dehydroepiandrosterone sulfate; LR: likelihood ratio; GB2: generalized beta of the second kind; GG: generalized gamma; HbA1c: glycated hemoglobin; SM: Singh–Maddala. For each biomarker, bold *p*‐values highlight those models that we are not able to reject the null hypothesis of restrictions being valid, according to both the LR and Wald tests, compared to the GB2 or GG models.

Table 2 shows that AIC and BIC results are in accordance with the tests of Table 1. For all biomarkers, linear regressions estimated by OLS perform worse than each of the four‐ and three‐parameter and most of the more parsimonious distributions. For fibrinogen, GB2 and SM perform best according to AIC and BIC criteria, with the latter showing the best performance. GB2 outperforms all the competing distributions regarding HbA1c. Although the B2 and SM distributions exhibit the best performance for the cholesterol ratio and DHEAS, GB2 is ranked the second best.

**Table 2:** AIC and BIC for each model

	Fibrinogen		Hba1c		Cholesterol ratio		DHEAS	
	AIC	BIC	AIC	BIC	AIC	BIC	AIC	BIC
GB2	**20,866**	20,948	**72,138**	**72,219**	**39,175**	39,257	**53,800**	53,889
B2	21,221	21,296	76,134	76,371	**39,173**	**39,249**	53,897	53,979
SM	**20,865**	**20,939**	72,329	72,404	39,432	39,506	**53,798**	**53,880**
Dagum	20,872	20,947	72,927	73,001	39,315	39,390	53,855	53,937
Fisk	20,883	20,950	73,563	73,629	39,482	39,549	54,149	54,223
Lomax	51,843	51,910	112,182	112,249	59,542	59,624	61,959	62,040
GG	21,204	21,278	74,986	75,060	39,180	39,270	53,927	54,016
Lognormal	21,502	21,569	77,305	77,372	39,306	39,373	54,407	54,482
Gamma	21,219	21,287	79,049	79,116	39,867	39,934	53,942	54,016
Weibull	22,804	22,871	88,676	88,743	42,443	42,518	54,640	54,715
Exponential	51,841	51,900	112,180	112,239	59,540	59,615	61,957	62,031
OLS	21,500	21,558	84,119	84,178	42,875	42,950	58,371	58,446

*Note*. AIC: Akaike information criteria; BIC: Bayesian information criteria; B2: beta of the second kind; DHEAS: dehydroepiandrosterone sulfate; GB2: generalized beta of the second kind; GG: generalized gamma; HbA1c: glycated hemoglobin; OLS: ordinary least squares; SM: Singh–Maddala. For each biomarker, bold values highlight those models that exhibit the best performance according to AIC and BIC.

**Figure 2:**
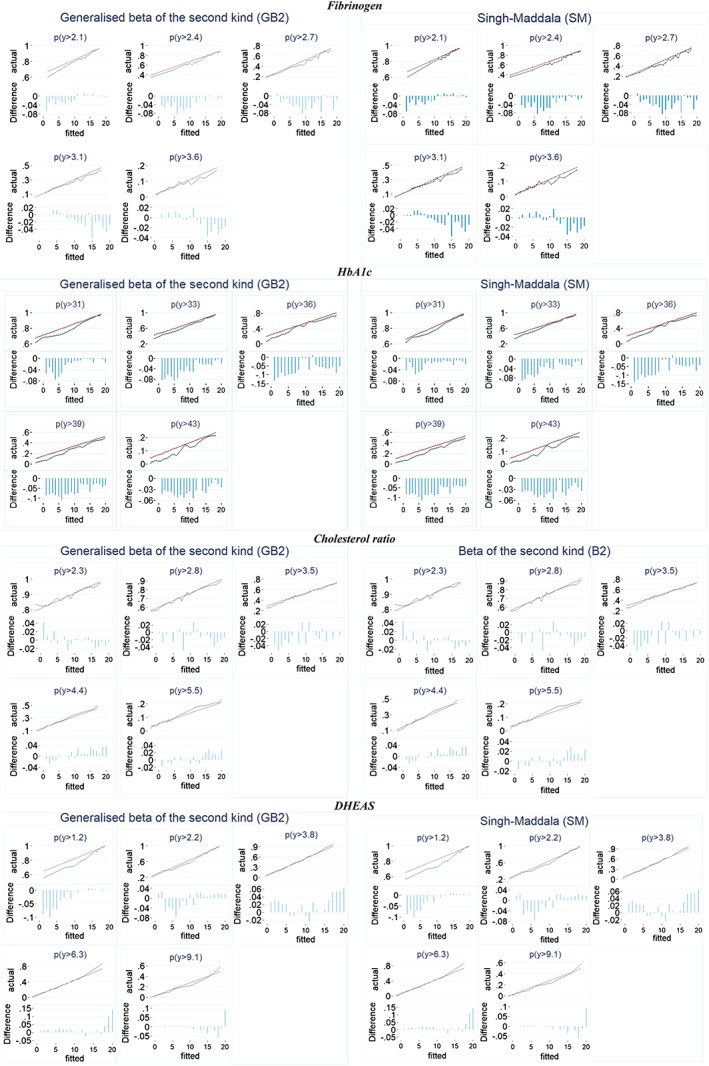
Actual versus fitted tail probabilities. DHEAS: dehydroepiandrosterone sulfate; HbA1c: glycated hemoglobin [Colour figure can be viewed at http://wileyonlinelibrary.com]

Figure 2 presents the conditional tail probabilities (at *k* equal to 10th, 25th, 50th, 75th, and 90th quantile) and spike plots of the actual‐fitted difference (bias) for the GB2 distribution, and its nested cases exerted the best performance for each biomarker (Table 2). Specifically, 20‐quantiles of the fitted values from these models are used to split the sample to calculate within‐quantiles means of actual [*P*(*y* > *k*)] and predicted [*P*(*y* > *k*| *X*)] probabilities.

There are limited differences in the predictive ability of the more parsimonious distributions compared with GB2, confirming previous evidence that a flexible distribution is not a substitute for finding the correct distribution (Jones et al., 2014). GB2 performs reasonably well at predicting tail probabilities, although there are some disparities at the very high fibrinogen levels (90th quantile) and HbA1c above the pre‐diabetes threshold (HbA1c ≥ 42).

## 5 CONCLUSION

We illustrate the comparative performance of a set of more flexible parametric distributions: the GB2, GG, and their nested and limiting cases for a set of biomarkers. Although some of the three‐parameter distributions nested within the GB2 (mainly the B2 and SM) outperform the latter in most of the biomarkers considered, GB2 can be used as a guide for choosing among competing distributions; a potentially useful message for applied researchers given that different biomarkers follow different distributions. The linear models estimated by OLS are dominated by almost all the competitive models. GB2 performs well at predicting biomarkers' tail probabilities, although with some disparities at the very high levels of fibrinogen and HbA1c.

## CONFLICT OF INTEREST

None.

## Appendix

**Table A1:** Descriptive statistics

Biomarker	Mean	Median	Standard deviation	Skewness	Kurtosis	Minimum	Maximum	Sample size
Fibrinogen (g/L)	2.79	2.70	0.59	0.47	3.82	0.40	5.50	12,811
HbA1c (mmol/mol)	37.25	36.00	8.19	4.17	31.15	12	133.0	12,153
Cholesterol ratio	3.74	3.46	1.36	1.42	6.43	1.16	13.67	12,865
DHEAS (μmol/L)	4.62	3.80	3.24	1.29	5.11	0.20	25.30	12,809

*Note*. DHEAS: dehydroepiandrosterone sulfate; HbA1c: glycated hemoglobin.

**Table A2:** Estimated parameters from the GB2 and the GG models

Biomarker	GB2			GG	
	α	*p*	*q*	κ	Ln(σ)
Fibrinogen	7.892 [7.017, 8.767]	1.104 [0.933, 1.275]	1.299 [1.063, 1.535]	0.267 [0.209, 0.326]	−1.606 [−1.621, −1.592]
HbA1c	42.986 [36.674, 49.298]	0.348 [0.287, 0.410]	0.198 [0.167, 0.230]	−0.461 [−0.555, −0.368]	−1.970 [−2.017, −1.924]
Cholesterol ratio	1.442 [0.777, 2.108]	23.345 [−9.920, 56.612]	6.611 [1.761, 11.463]	−0.246 [−0.290, −0.200]	−1.169 [−1.183, −1.157]
DHEAS	2.538 [2.202, 2.873]	1.036 [0.846, 1.225]	2.316 [1.717, 2.915]	0.446 [0.396, 0.495]	−0.615 [−0.631, −0.599]

*Note*. DHEAS: dehydroepiandrosterone sulfate; GB2: generalized beta of the second kind; GG: generalized gamma; HbA1c: glycated hemoglobin.
